# Long-term outcomes of drug-coated balloon treatment of calcified coronary artery lesions: a multicenter, retrospective, propensity matching study

**DOI:** 10.3389/fcvm.2023.1122290

**Published:** 2023-06-14

**Authors:** Yingguang Shan, Wenjie Lu, Zhanying Han, Sancong Pan, Xiangbing Li, Xi Wang, Liang Pan, Xule Wang, Xiaolin Zheng, Ran Li, Yanjun Zhou, Peng Qin, Qiangwei Shi, Shuai Zhou, Wencai Zhang, Sen Guo, Jing Qiu, Peisheng Zhang, Xiaofei Qin, Guoju Sun, Zhongsheng Qin, Zhenwen Huang, Chunguang Qiu

**Affiliations:** ^1^ Department of Cardiovascular Medicine, The First Affiliated Hospital of Zhengzhou University, Zhengzhou, China; ^2^ Department of Cardiovascular Medicine, Jincheng People’s Hospital, Jincheng, China; ^3^ Department of Geriatric Cardiology, The First Affiliated Hospital of Zhengzhou University, Zhengzhou, China; ^4^ Department of Cardiology, The Fifth Affiliated Hospital of Zhengzhou University, Zhengzhou, China

**Keywords:** coronary artery calcification, drug-coated balloon, *de novo*, PCI, propensity matching

## Abstract

**Background:**

Coronary artery calcification (CAC) is associated with high rates of restenosis and adverse clinical events after percutaneous coronary intervention (PCI) with drug-eluting stents (DES).

**Objectives:**

The aim of this study was to evaluate the long-term clinical outcomes of drug-coated balloon (DCB)-only treatment for *de novo* lesions with and without CAC.

**Methods:**

Patients with *de novo* coronary disease treated with the DCB-only strategy were retrospectively enrolled from three centers and categorized into a CAC group and a non-CAC group. The primary endpoint was the target lesion failure (TLF) rate during the 3-year follow-up. Secondary endpoints included the occurrence of major adverse cardiac events (MACEs), target lesion revascularization (TLR), cardiac death, myocardial infarction (MI) and any revascularization. Propensity score matching (PSM) was conducted to assemble a cohort of patients with similar baseline characteristics.

**Results:**

A total of 1,263 patients with 1,392 lesions were included, and 243 patients were included in each group after PSM. Compared with the non-CAC group, the incidence rates of TLF (9.52% vs. 4.94%, odds ratio [OR]: 2.080; 95% confidence interval [CI]: 1.083–3.998, *P* = 0.034) and TLR (7.41% vs. 2.88%, OR: 2.642; 95% CI: 1.206–5.787, *P* = 0.020) in the CAC group were higher. The incidence rates of MACE (12.35% vs. 7.82%, OR: 1.665; 95% CI: 0.951–2.916, *P* = 0.079), cardiac death (2.06% vs. 2.06%, OR: 0.995; 95% CI: 0.288–3.436, *P* = 0.993), MI (1.23% vs. 0.82%, OR: 2.505; 95% CI: 0.261–8.689, *P* = 0.652) and any revascularization (12.76% vs. 9.67%, OR: 1.256; 95% CI: 0.747–2.111, *P* = 0.738) were similar between groups.

**Conclusions:**

**CAC** increased the incidence of TLF and TLR without a substantial increase in the risk of MACE, cardiac death, MI, or any revascularization in patients treated with DCB-only angioplasty during the 3-year follow-up.

## 1. Introduction

Previous studies have shown that coronary artery calcification (CAC) at the target lesion site is associated with high rates of revascularization and adverse clinical events after percutaneous coronary intervention (PCI) with drug-eluting stent (DES) implantation (1, 2). Even second-generation DESs with thin-strut platforms and biocompatible or biodegradable polymers have been shown to reduce adverse outcomes in patients with moderate to severe CAC compared with early-generation DESs but are still unsatisfactory (3). Drug-coated balloons (DCBs) have been widely used to treat in-stent restenosis (ISR) (4). Recently, increasing evidence has shown that DCBs are effective for treating *de novo* coronary artery lesions, particularly small vessel disease (5). The potential use of DCBs as an alternative treatment for CAC lesions has attracted widespread interest among scholars, but their application in treating these lesions has rarely been reported.

To date, only a few studies have focused on DCB treatment of patients with CAC, but the results are inconsistent (6–10). This discrepancy may be due to the differences in inclusion criteria, operation strategies, and follow-up periods among studies, and all these studies were limited by small sample sizes. Thus, the safety and efficiency of DCB treatment in patients with CAC in real-world practice remain unclear. Therefore, we performed the present study for patients with *de novo* coronary artery disease treated using a DCB-only strategy and evaluated the 3-year clinical outcomes for patients with and without CAC.

## 2. Methods

### 2.1. Study population

**Figure 1:**
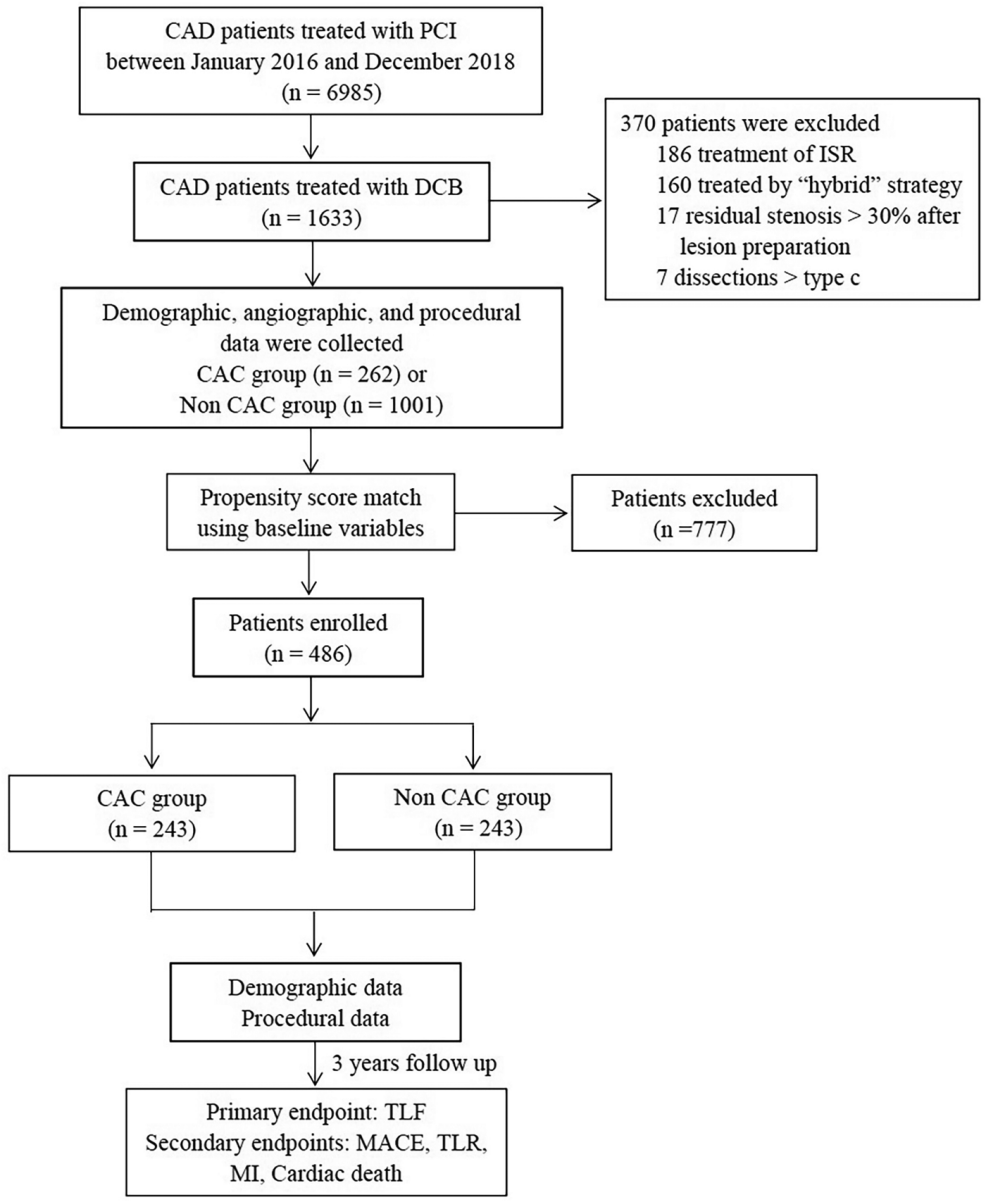
Study flowchart. CAD, Coronary Artery Disease; PCI, Percutaneous Coronary Intervention; DCB, Drug Coated Balloon; ISR, In-Stent Restenosis; TLF, Target Lesion Failure; MACE, Major Adverse Cardiac Events; TLR, Target Lesion Revascularization; MI, Myocardial Infarction.

From January 2016 to December 2018, 1,392 lesions in 1,263 consecutive patients treated with the DCB angioplasty-only strategy at three centers were retrospectively reviewed. According to a modified scheme of the American College of Cardiology and American Heart Association, lesion calcification was assessed angiographically and classified into none or mild, moderate (defined as radiopaque densities noted during cardiac motion involving only 1 side of the vascular wall), and severe (defined as radiopaque densities noted without cardiac motion generally involving both sides of the arterial wall). Patients with moderate or severe calcification were included in the CAC group, while the others were included in the non-CAC group. The exclusion criteria were ISR lesions, “hybrid” treated lesions (one lesion treated with DCB and DES), >30% residual stenosis or ≥type C dissection after lesion preparation, unstable hemodynamics, and life expectancy less than one year (Figure 1).

Dual antiplatelet therapy (DAPT) was administered before the intervention and continuously used for at least three months for stable angina and six months for ACS after the procedure by patients who underwent DCB-only treatment. The duration of DAPT for those patients who were simultaneously treated with DCB and DES was based on the established guidelines (11). Ethical approval for the study was granted by the First Affiliated Hospital of Zhengzhou University Institutional Review Board/Ethics Committee. Written informed consent was obtained from all patients. A common electronic case report form was used for data collection.

### 2.2. Operation procedure

The operation procedure was performed as reported previously (12). Briefly, a noncompliant balloon, dual-wire balloon, scoring balloon or cutting balloon was used for predilatation with a balloon-to-vessel ratio of 0.8–1.0. Rotational atherectomy (RA) was performed if the lesion was not crossable by any balloon, not adequately dilatable, or the DCB could not be delivered to the lesion despite complete balloon expansion. DCB angioplasty was used to treat lesions without flow-limiting dissection [<type C according to the NHLBI (National Heart, Lung, and Blood Institute) classification] and severe residual stenosis (>30%) (13). The length of the DCB used was at least 2 mm longer than the lesion at both edges to avoid a geographic mismatch. The diameter of the DCB was selected with a balloon-to-vessel ratio of 0.8–1.0. Paclitaxel-coated balloons (SeQuent Please; B Braun, Melsungen, Germany) were used in the present study at an inflation pressure of >7 bars for more than 30 s (usually 60 s).

### 2.3. Clinical endpoints

The follow-up for patients was conducted through telephone outpatient visits. The primary endpoint of this study was the target lesion failure (TLF) rate during the 3-year follow-up, a composite outcome of cardiac death, target vessel myocardial infarction (MI), and target lesion revascularization (TLR). Secondary endpoints included the occurrence of TLR, MI, cardiac death, major adverse cardiac events (MACEs, defined as the composite outcome of cardiac death, MI, and target vessel revascularization) and any revascularization.

### 2.4. Statistical analysis

Data were analyzed using SPSS version 20.0 and GraphPad Prism version 9 software. Categorical variables are presented as frequencies (percentages) and were tested using the chi-square test or Fisher's exact test. Continuous variables are presented as the means ± SDs and were analyzed using an independent *t*-test or the Mann‒Whitney nonparametric test. One-to-one propensity score matching (PSM) was used to select patients with comparable baseline data. Eight variables (age, sex, diabetes mellitus, hypertension, hyperlipidemia, prior stroke, renal insufficiency, acute coronary syndrome, and prior PCI) were included in the propensity score matching model using greedy nearest neighbor matching without replacement and a caliper of 0.02. Outcomes were compared using the log-rank test and presented as Kaplan‒Meier curves. All *P*-values were two-sided, and *P* < 0.05 was defined as statistically significant.

## 3. Results

### 3.1. Characteristics of patients

A total of 1,263 patients with *de novo* lesions who were treated with the DCB-only strategy were included. Within this patient population, 262 individuals (20.7%) were identified as having moderate or severe calcification. After 1:1 matching, 243 patients were selected for each group. Patients in the CAC group were older, and the proportions of females, patients with diabetes mellitus, hypertension, hyperlipidemia, prior stroke or TIA, renal insufficiency, and a prior PCI history were higher than those in the non-CAC group (all *P* < 0.05). Other clinical parameters, such as a history of smoking, family history of CAD, ACS, prior MI, prior CABG, LVEF, and other vessels treated using DES, did not differ significantly between groups. Parameters with significant differences between the groups were reduced after propensity score matching (Table 1).

**Table 1:** Clinical characteristics between groups before and after propensity score matching.

Variables	All patients (*n* = 1,263)			Propensity matched sample (*n* = 486)		
	CAC group (*n* = 262)	Non CAC group (*n* = 1,001)	*P-*value	CAC group (*n* = 243)	Non CAC group (*n* = 243)	*P-*value
Age (years)	63.45 ± 9.72	58.36 ± 11.10	<0.001	63.27 ± 9.56	62.36 ± 10.55	0.322
Male [*n*, (%)]	168 (64.12)	726 (72.53)	0.008	157 (64.61)	160 (65.84)	0.775
Diabetes mellitus [*n*, (%)]	80 (30.53)	214 (21.38)	0.002	75 (30.86)	72 (29.63)	0.767
Hypertension [*n*, (%)]	153 (58.40)	513 (51.25)	0.03	142 (58.44)	137 (56.38)	0.646
Hyperlipidemia [*n*, (%)]	85 (32.44)	247 (24.68)	0.011	79 (32.51)	70 (28.81)	0.376
History of smoking [*n*, (%)]	81 (30.92)	341 (34.07)	0.336	75 (30.86)	72 (29.63)	0.767
Family history of CAD [*n*, (%)]	43 (16.41)	166 (16.58)	0.947	40 (16.46)	38 (15.64)	0.805
Prior stroke or TIA [*n*, (%)]	45 (17.17)	121 (12.09)	0.030	41 (16.87)	38 (15.64)	0.712
Renal insufficiency [*n*, (%)]	24 (9.16)	41 (4.10)	0.001	22 (9.05)	18 (7.41)	0.509
Acute coronary syndrome [*n*, (%)]			0.775			0.928
Unstable angina [*n*, (%)]	166 (63.36)	620 (61.94)		156 (64.20)	155 (63.78)	
NSTEMI [*n*, (%)]	19 (7.25)	81 (8.09)		16 (6.60)	18 (7.41)	
STMI [*n*, (%)]	14 (5.34)	45 (4.50)		12 (4.94)	13 (5.35)	
Prior MI [*n*, (%)]	20 (7.63)	95 (9.49)	0.352	16 (6.58)	19 (7.82)	0.599
Prior PCI [*n*, (%)]	54 (20.61)	124 (12.39)	0.001	50 (20.58)	50 (20.58)	0.278
Prior CABG [*n*, (%)]	9 (3.44)	19 (1.90)	0.133	8 (3.29)	7 (2.88)	0.793
HbA1c (%)	6.53 ± 2.88	6.23 ± 2.42	0.087	6.52 ± 2.87	6.44 ± 2.53	0.744
Triglycerides (mmol/L)	1.23 ± 1.02	1.12 ± 0.91	0.090	1.23 ± 1.02	1.18 ± 0.88	0.563
HDL-c (mmol/L)	1.33 ± 1.21	1.42 ± 1.44	0.353	1.33 ± 1.21	1.38 ± 1.33	0.665
LDL-c (mmol/L)	2.11 ± 1.32	2.04 ± 1.55	0.503	2.11 ± 1.33	2.09 ± 1.46	0.875
Statin [*n*, (%)]	260 (99.24)	989 (98.80)	0.789	241 (99.18)	240 (98.77)	1.000
ACEI/ARB [*n*, (%)]	157 (59.92)	572 (57.14)	0.417	144 (59.25)	138 (56.79)	0.581
Beta-blocker [*n*, (%)]	167 (63.74)	601 (60.04)	0.275	153 (62.96)	142 (58.44)	0.307
Duration of DAPT (months)	10.63 ± 3.84	10.42 ± 3.44	0.412	10.61 ± 3.81	10.53 ± 3.52	0.810
LVEF (%)	59.76 ± 5.32	59.40 ± 6.82	0.373	59.82 ± 5.45	59.32 ± 6.39	0.243
Other vessels treated by DES [*n*, (%)]	109 (41.60)	372 (37.16)	0.188	101 (41.56)	95 (39.09)	0.589

CAD, Coronary Artery Disease; TIA, Transient Ischemic Attack; NSTEMI, Non-ST-segment Elevation Myocardial Infarction; STEMI ST-Segment Elevation Myocardial Infarction; MI, Myocadiac Infarction; PCI, Percutaneous Coronary Intervention; CABG, Coronary Artery Bypass Grafting; LVEF, Left Ventricular Ejection Fraction; DES, Drug Eluting Stent; HbA1c, Glycosylated hemoglobin; HDL-c, High Density Dipoprotein cholesterol; LDL-c, Low Density Lipoprotein cholesterol; ACEI/ARB, Angiotensin Converting Enzyme Inhibitor / Angiotensin Receptor antagonist; DAPT, duration of dual antiplatelet therapy.

### 3.2. Lesion characteristics and procedural data

The procedural characteristics before and after propensity score matching are shown in Table 2. Notably, 1,392 lesions were treated before matching, of which 252 (17.5%) were CAC lesions. After matching, 513 lesions remained: 252 in the CAC group and 261 in the non-CAC group. In the matched cohorts, the differences in lesion morphology and procedural data between groups were comparable. Lesion predilatation with different balloons (semicompliant balloon, NSE, cutting balloon, dual-wire balloon, and noncompliant balloon) was performed for all lesions. For the CAC group and non-CAC group, the number of DCBs used per lesion was 1.06 ± 0.27 and 1.05 ± 0.24, the mean DCB diameters were 2.70 ± 0.41 mm and 2.69 ± 0.46 mm, the length of the DCB balloon used per lesion was 23.51 ± 10.57 mm and 22.98 ± 10.69 mm, and the inflation pressures were 8.24 ± 1.29 atm and 8.34 ± 1.41 atm, respectively. All patients were advised to reexamine angiography six months to one year later and to be hospitalized at any time for reexamination of angiography if they had ischemia symptoms.

**Table 2:** Procedural characteristics before and after propensity score matching.

Variables	All lesions (*n* = 1,392)			Propensity matched sample (*n* = 513)		
	CAC group (*n* = 272)	Non CAC group (*n* = 1,120)	*P-*value	CAC group (*n* = 252)	Non CAC group (*n* = 261)	*P-*value
Treated vessel			0.232			0.395
Left main coronary artery [*n*, (%)]	1 (0.37)	4 (0.36)		1 (0.40)	0 (0.00)	
Left anterior descending coronary artery [*n*, (%)]	124 (45.59)	467 (41.68)		116 (46.03)	110 (42.15)	
Left circumflex coronary artery [*n*, (%)]	112 (41.18)	437 (39.00)		105 (41.67)	107 (41.00)	
Right coronary artery [*n*, (%)]	34 (12.50)	205 (18.34)		29 (11.51)	41 (15.71)	
Number of lesions treated by DCB (per patient)			0.005			0.157
1	253 (96.56)	895 (89.41)		235 (96.71)	226 (93.00)	
2	8 (3.05)	95 (9.49)		7 (2.88)	16 (6.58)	
3	1 (0.38)	9 (0.90)		1 (0.41)	1 (0.41)	
4	0 (0.00)	2 (0.20)		0 (0.00)	0 (0.00)	
Lesion type						
Total occlusion [*n*, (%)]	45 (16.54)	124 (11.07)	0.013	42 (16.67)	36 (13.79)	0.365
Intracoronary thrombus [*n*, (%)]	2 (0.74)	6 (0.54)	0.696	2 (0.79)	1 (0.35)	0.542
Diffuse vessel disease [*n*, (%)]	70 (25.74)	211 (18.84)	0.011	66 (26.19)	62 (23.75)	0.524
Ostial lesion [*n*, (%)]	50 (18.38)	178 (15.89)	0.320	46 (18.25)	57 (21.84)	0.311
Bifurcation lesion [*n*, (%)]	134 (49.26)	411 (36.70)	<0.001	124 (49.21)	107 (41.00)	0.062
Lesion preparation [*n*, (%)]	272 (100.00)	1,120 (100.00)	1.000	251 (100.00)	262 (100.00)	1.000
Semi-compliant balloon [*n*, (%)]	202 (74.26)	724 (64.64)	0.003	187 (74.21)	186 (71.26)	0.455
NSE [*n*, (%)]	88 (32.35)	340 (30.36)	0.522	85 (33.73)	70 (26.82)	0.088
Cutting balloon [*n*, (%)]	50 (18.38)	184 (16.43)	0.440	48 (19.05)	40 (15.33)	0.264
DWB [*n*, (%)]	3 (1.10)	17 (1.52)	0.606	3 (1.19)	8 (3.07)	0.143
Noncompliant balloon [*n*, (%)]	74 (27.21)	199 (17.77)	<0.001	69 (27.38)	54 (20.69)	0.076
RA [*n*, (%)]	32 (11.76)	0 (0.00)	<0.001	29 (11.51)	0 (0.00)	<0.001
Number of DCBs used (per lesion)	1.06 ± 0.27	1.04 ± 0.22	0.212	1.06 ± 0.27	1.05 ± 0.24	0.564
Mean DCB diameter (mm)	2.70 ± 0.42	2.70 ± 0.47	0.854	2.70 ± 0.41	2.69 ± 0.46	0.753
Length of DCB balloon (per lesion, mm)	23.44 ± 10.41	22.53 ± 9.31	0.132	23.51 ± 10.57	22.98 ± 10.69	0.540
Inflation pressure (bar)	8.24 ± 1.28	8.20 ± 1.27	0.635	8.24 ± 1.29	8.34 ± 1.41	0.380
TIMI flow grade						
Before procedure	2.43 ± 1.13	2.53 ± 1.04	0.163	2.42 ± 1.14	2.46 ± 1.07	0.660
After procedure	2.91 ± 0.36	2.93 ± 0.25	0.296	2.92 ± 0.32	2.93 ± 0.31	0.926

NSE, non-slip element; DWB, Dual wire balloon; RA, Rotational Atherectomy; DCB, Drug Coated Balloon. Diffuse vessel disease was defined as “lesion length >25 mm”; Ostial lesion was defined as “lesion located within 3 mm of the ostial”; Bifurcation lesion was defined as “Medina 1,0,1, 0,1,1 or 1,1,1”.

### 3.3. In-hospital events

In the present study, 3 (0.24%) of the 1,263 patients experienced in-hospital acute ischemic events, and all events occurred within 3 h after the operation. Emergency angiography showed that 2 patients had a target vessel TIMI flow of grade 1, and 1 patient had target vessel occlusion. Intravascular ultrasound revealed severe dissections and hematomas in the target lesions. DES implantation was performed in these patients, and none of the 3 patients had a Q-wave myocardial infarction. Interestingly, all 3 patients were from the non-CAC group.

### 3.4. Clinical outcomes

**Figure 2:**
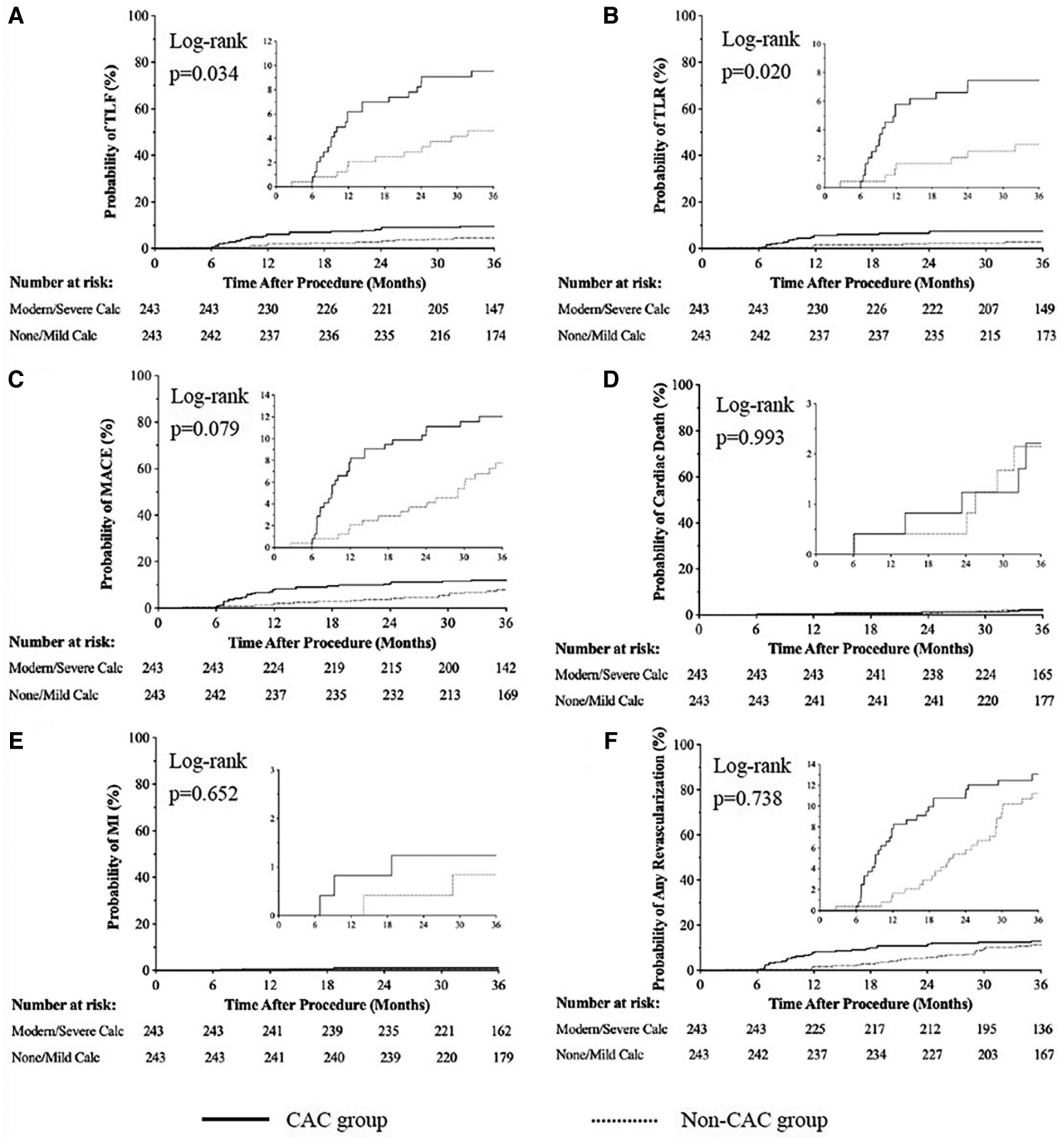
Cumulative risks of the study outcomes [TLF (**A**), TLR (**B**), MACE (**C**), cardiac death(**D**), MI (**E**), Any revascularization (**F**)] at 3-year follow up. Cardiac Events; MI, Myocardiac Infarction.

In the overall population (Table 3), 1,130 of 1,263 patients (89.47%) were available for 3 years of follow-up. The incidence rates of TLF were 9.52% and 4.60% in CAC and non-CAC patients, respectively (OR: 2.346, 95% CI: 1.293–4.253; log rank *P* < 0.001). After propensity matching (Table 3 and Figure 2A), the difference in the incidence rates of TLF was still significant between the groups (9.52% vs. 4.94% for the CAC group and non-CAC group, respectively; OR: 2.080, 95% CI: 1.082–3.998; log rank *P* = 0.034).

**Table 3:** Clinical outcomes in the propensity score-matched cohort at three-year follow-up.

Variables	All patients (*n* = 1,263)				Propensity matched sample (*n* = 486)			
	CAC group (*n* = 262)	Non CAC group (*n* = 1,001)	Odds ratio (95% CI)	*P-*value	CAC group (*n* = 243)	Non CAC group (*n* = 243)	Odds ratio (95% CI)	*P–*value
TLF [*n*, (%)]	25 (9.54)	46 (4.60)	2.421 (1.392, 4.341)	<0.001	24 (9.52)	12 (4.94)	2.080 (1.082, 3.998)	0.034
TLR [*n*, (%)]	19 (7.25)	27 (2.70)	2.674 (1.139, 5.414)	<0.001	18 (7.41)	7 (2.88)	2.642 (1.206, 5.787)	0.020
MACE [*n*, (%)]	32 (12.21)	68 (6.79)	1.942 (1.107, 3.263)	0.003	30 (12.35)	19 (7.82)	1.665 (0.951, 2.916)	0.079
Cardiac death [*n*, (%)]	5 (1.91)	14 (1.40)	1.424 (0.348, 5.763)	0.578	5 (2.06)	5 (2.06)	0.995 (0.288, 3.436)	0.993
MI [*n*, (%)]	4 (1.53)	10 (1.00)	1.457 (0.422, 4.977)	0.725	3 (1.23)	2 (0.82)	1.505 (0.261, 8.689)	0.652
Any revascularization [*n*, (%)]	34 (12.98)	78 (7.79)	1.863 (1.347, 3.122)	0.010	31 (12.76)	26 (9.67)	1.256 (0.747, 2.111)	0.738

TLF, Target Lesion Failure; TLR, Target Lesion Revascularization; MACE, Major Adverse Cardiac Events; MI, Myocardial Infarction.

For the propensity matched sample, Kaplan‒Meier analysis (Table 3 and Figures 2B–2F) revealed that the incidence rates of TLR were higher in patients with calcified lesions than in patients with noncalcified lesions (7.41% vs. 2.88%, OR: 2.642, 95% CI: 1.206, 5.787; log rank *P* = 0.020). Meanwhile, the incidence rates of MACE (12.35% vs. 7.82%, OR: 1.665, 95% CI: 0.951, 2.916; log rank *P* = 0.079), cardiac death (2.06% vs. 2.06%, OR: 0.995, 95% CI: 0.288, 3.436; log rank *P* = 0.993), MI (1.23% vs. 0.82%, OR: 1.505, 95% CI: 0.261, 8.689; log rank *P* = 0.652), and any revascularization (12.76% vs. 9.67%, OR: 1.256, 95% CI: 0.747, 2.111; log rank *P* = 0.738) were not significantly different between groups.

Additionally, we analyzed 17 patients who were treated with DCBs but were excluded due to unsatisfactory residual stenosis (30%–50%). The incidence of TLF during the 3-year follow-up was 14.29% (1 of 7 patients) in the CAC group and 10.00% (1 of 10 patients) in the non-CAC group.

### 3.5. Sensitivity and subgroup analysis

In the whole population, TLF exhibited good consistency before and after PSM. The incidence rates of TLR (OR: 2.674, 95% CI: 1.139–5.414), MACE (OR: 1.942, 95% CI: 1.107–3.263), cardiac death (OR: 1.494, 95% CI: 0.478–4.679), and any revascularization (OR: 1.688, 95% CI: 1.047–2.723) in the CAC group were higher than those in the non-CAC group (all *P* < 0.05, Table 3). Additionally, a binary logistic regression analysis of TLF in the overall population revealed that CAC increased the TLF risk (OR: 1.887, 95% CI: 1.137–4.776, *P* = 0.032).

After PSM, patients were divided into ACS and non-ACS groups for subgroup analysis. For patients in the ACS subgroup, the incidence rates of TLF (10.87% vs. 4.84%, OR: 2.310, 95% CI: 1.115–4.785) and TLR (8.15% vs. 2.69%, OR: 2.872, 95% CI: 1.565–6.182) in the CAC group were higher than those in the non-CAC group (all *P* < 0.05), while the incidence of MACE (12.50% vs. 8.06%, OR: 1.875, 95% CI: 0.876–3.476), cardiac death (2.17% vs. 2.15%, OR: 0.996, 95% CI: 0.285–3.476), and any revascularization (14.13% vs. 12.37%, OR: 1.338, 95% CI: 0.667–1.998) were similar between groups (all *P *> 0.05). On the other hand, for patients in the non-ACS subgroup, the incidence of TLF (6.78% vs. 5.26%, OR: 1.169, 95% CI: 0.265–5.160), TLR (5.08% vs. 3.51%, OR: 1.335, 95% CI: 0.231–7.730), MACE (11.86% vs. 7.02%, OR: 2.159, 95% CI: 0.658–7.085), cardiac death (1.69% vs. 1.75%, OR: 0.855, 95% CI: 0.053–13.78), and any revascularization (8.47% vs. 5.26%, OR: 1.807, 95% CI: 0.451-) were similar between the CAC group and the non-CAC group (all *P *> 0.05).

## 4. Discussion

The present study retrospectively analyzed patients with *de novo* calcified coronary artery lesions treated with a DCB-only strategy. The incidence rates of TLF and TLR after DCB treatment in the CAC group were higher than those in the non-CAC group during the three-year follow-up. However, the incidence rates of MACEs, cardiac death, myocardial infarction, and any revascularization between the groups were similar. To our knowledge, this report is the first to document DCB-only treatment for *de novo* CAC lesions in the real world. Moreover, our results are based on propensity score matching with the largest sample investigated to date. This study provides new evidence for DCB treatment of *de novo* CAC lesions, suggesting that DCB is safe and effective for these lesions.

Due to the unique characteristics of calcified lesions, such as irregular lesion surfaces, a resistant plaque burden and heavily calcified stenosis, they are difficult to adequately dilate, which may contribute to failure of stent delivery or incomplete stent expansion (1, 2, 14). Paulet (3) investigated 6,211 patients with moderate or severe CAC who were treated with DES; the incidence of TLF was 19.8% in patients treated with first-generation DES and 14.8% in patients treated with second-generation DES at the 5-year follow-up. However, as a stentless strategy, DCBs have various advantages over DESs in treating *de novo* CAC lesions. The antiproliferative drug distribution was much more uniform, and the concentrations released were higher with DCB treatment, which is more conducive to inhibiting inflammatory reactions, resulting in better positive remodeling. Moreover, even if target lesion restenosis occurs, DCB or DES may be implanted again, or even CABG, providing additional opportunities for long-term treatment.

Various studies have shown that the restenosis rate is very high 10 years after DES implantation (15, 16). Although DCBs have been used for ISR treatment, their efficacy is inferior to that of treating *de novo* lesions (12). In recent years, DCBs have developed rapidly from ISR to *de novo* lesions. A number of clinical studies have shown that DCBs have good efficacy in the treatment of *de novo* lesions, including small vessels and bifurcation lesions (5, 17–19). However, few reports have described the application of DCBs to treat *de novo* CAC lesions. With increased experience, some scholars reported that DCBs could be applied to calcified lesions after RA, which provided a theoretical and practical basis for the application of DCBs as a treatment for calcified lesions in the present study. Tuomas (6) reported the treatment of 65 patients with CAC and a high risk of bleeding using RA followed by DCB in 2017, and the incidence of MACEs was 20% during the 2-year follow-up. Subsequently, Taito (7) investigated the acute and mid-term efficacy of DCB following RA for 190 complex CAC lesions; angiographic restenosis was observed in 17.8% of RA + DCB-treated lesions, with a mean late lumen loss of 0.23 ± 0.69 mm and a TLR rate of 16.4%. Moreover, Jun (8) reported thirty patients (34 lesions) undergoing RA/DCB for *de novo* CAC lesions with a mean follow-up period of 13.1 months, and 6 deaths (2 sudden deaths, 1 cardiac death, 3 noncardiac deaths), 2 strokes, and 2 target lesion revascularizations occurred. Although these data were obtained from single-armed, retrospective, small sample studies, the results show that DCB is a safe and effective treatment for patients with CAC undergoing RA. Additional data on treating CAC with DCB were derived from an observational, retrospective study (9); 123 consecutive patients (166 lesions) with *de novo* CAC undergoing an image-guided rotational atherectomy (iRA) followed by DCB (DCB-iRA; 54 patients, 68 lesions) or implantation of new generation drug-eluting stents (nDES-iRA; 69 patients, 98 lesions) at a single center were analyzed. Follow-up angiography was obtained at >6 months, and the median clinical follow-up was 732 days. The TLR and TVR rates in patients treated with DCB-iRA and nDES-iRA were similar: 15.6% vs. 16.3% (*P* = 0.99) and 15.6% vs. 23.3% (*P* = 0.38), respectively. In 41 well-matched lesion pairs after propensity score matching, the cumulative incidence rates of TLR and TVR in patients treated with DCB-iRA and nDES-iRA were 12.9% vs. 16.3% (*P* = 0.70) and 12.9% vs. 26.1% (*P* = 0.17), respectively. On the other hand, Ryuta (10) reported 81 patients (46 in the CAC group and 35 in the non-CAC group) who were treated with DCBs, and the incidence rates of TLR and MACEs were comparable between the groups, in contrast to the present study. The possible explanations for the difference are provided below. First, the diameter of the target lesions in the patients enrolled in the current study was relatively large, and the problems of the target vessels tended to cause corresponding clinical events. Second, the pretreatment of the patients in the two studies was different. The rate of RA application in the calcified lesion group was 84.0% in the study by Ryuta but only 11.5% in our current study. Third, the number of cases in the study by Ryuta was obviously small, which may lead to a lack of statistical validity.

Overall, previous studies have explored PCI therapy for CAC lesions, and the results vary depending on the population, lesions, surgical protocol, and duration of follow-up. Regardless of the differences among the studies mentioned above, we found that all the studies focused on CAC patients treated with RA followed by DCB implantation. Only a fraction of CAC lesions need RA, and previous studies do not reflect the real-world usage of DCBs to treat CAC lesions. The present study compensates for the shortcomings of previous studies and documents the long-term clinical results for the DCB-only strategy to treat CAC lesions. Although our study showed that the occurrence of TLR and TLF in the CAC group was significantly higher than that in the non-CAC group, we postulate that this result is acceptable because the results from calcified lesions treated with DES may be worse. Although we were unable to draw this conclusion in the present study, the incidence rates of TLF and TLR in this study were relatively low compared with those in other studies investigating stent implantation for CAC lesions. Of course, further head-to-head randomized clinical trials are needed to verify whether DCBs have an advantage over DESs in treating these lesions. Moreover, subgroup analysis showed that in ACS patients, the occurrence of TLR and TLF in the CAC group was significantly higher than that in the non-CAC group, while no similar results were found in non-ACS patients. Large-sample randomized controlled trials are needed to further verify this result.

The safety of DCB application in *de novo* lesions, especially large vessels, is a wide concern (17). Acute vascular occlusion caused by dissection may lead to serious consequences. In the present study, only 0.24% of patients in the whole cohort experienced acute ischemic events within 3 h after the procedure, and all patients recovered TIMI grade 3 blood flow after stent implantation with non-Q wave myocardial infarction. Interestingly, no patients in the CAC group experienced acute events, potentially due to the very low incidence of acute events, the inadequate sample size, and the fact that operators may be more cautious when treating calcified lesions. Thus, based on our findings, DCB treatment is safe for *de novo* calcified and noncalcified lesions after adequate lesion preparation.

In addition, of the 17 patients who were excluded from this study and received DCB treatment for 30%–50% residual stenosis, 7 had moderate to severe calcification. For the reasons of residual stenosis, three of the seven patients were due to severe local calcified nodules, leading poor expansion of the pre-dilation balloon, and stent implantation was easy to form poor adherence and cause stent thrombosis; The other four patients had bifurcation lesions, residual stenosis were due to elastic recoil of the branch ostial even after sufficient pre-dilation, stent implantation may affect the other branch, so we used DCB. Although the incidence of TLF was higher than that of patients with less than 30% residual stenosis (14.29% vs. 9.52%), this result was acceptable compared to those who received DES treatment. Current guidelines do not recommend the use of DCBs for patients with more than 30% residual stenosis after predilation (11), but some CAC lesions with 30% to 50% residual stenosis are still treated with DCBs. In the real world, even after RA, some patients with calcified lesions still exhibit inadequate expansion, and stent implantation in these lesions usually results in poor expansion, leading to higher rates of acute thrombosis and late restenosis. Therefore, clinicians often focus on the PCI strategy of these patients. Combined with the data reported in the present study, we propose that DCB implantation may be another reasonable choice for these patients. Of course, the sample size of these patients was limited in the present study, and more clinical studies are needed in the future. Nevertheless, our limited data provide a new perspective for interventional treatment for patients with this condition.

In summary, the DCB-only strategy is a safe and effective treatment for *de novo* CAC. This study has accumulated more experience for the treatment of *de novo* CAC lesions, but it also has certain limitations. First, as a retrospective study, a certain bias existed in the inclusion of patients, despite propensity score matching. Second, approximately forty percent of patients in both groups received DES implantation, which may affect the incidence of clinical events. Third, compared to other studies, the application of RA in the CAC group was lower in our study, and we have not determined whether this difference might affect clinical outcomes. In addition, our study used angiography to assess the severity of calcification, previous study (20) shows coronary angiography can detected 31% of 1-quadrant calcium, 54% of 2-quadrant calcium, 75% of 3-quadrant calcium, and 83% of 4-quadrant calcium seen by IVUS, and angiography could not distinguish calcification located in the intima or the intima, therefore, the results in our study may not be as accurate as intravascular imaging. Unfortunately, to date, there are no studies on the treatment of calcified lesions with DCB guided by intravascular imaging. Finally, our study compared the clinical outcomes between patients with and without calcification, and the results do not answer the question of whether DCB is superior to DES for the treatment of patients with CAC. Thus, additional prospective randomized clinical trials are needed to overcome these limitations.

## 5. Conclusions

Compared with non-CAC lesions, CAC in target lesions increased the incidence of TLF and TLR upon DCB angioplasty in the present study. However, the incidence rates of MACEs, cardiac death, MI, and any revascularization were similar between the groups.

## Data Availability

The original contributions presented in the study are included in the article, further inquiries can be directed to the corresponding author.
